# Optical-helicity-driven magnetization dynamics in metallic ferromagnets

**DOI:** 10.1038/ncomms15085

**Published:** 2017-04-18

**Authors:** Gyung-Min Choi, André Schleife, David G. Cahill

**Affiliations:** 1 Center for Spintronics, Korea Institute of Science and Technology, Seoul 02792, Korea; 2 Department of Materials Science and Engineering and Materials Research Laboratory, University of Illinois, Urbana, Illinois 61801, USA

## Abstract

Recent observations of switching of magnetic domains in ferromagnetic metals by circularly polarized light, so-called all-optical helicity dependent switching, has renewed interest in the physics that governs the interactions between the angular momentum of photons and the magnetic order parameter of materials. Here we use time-resolved-vectorial measurements of magnetization dynamics of thin layers of Fe, Ni and Co driven by picosecond duration pulses of circularly polarized light. We decompose the torques that drive the magnetization into field-like and spin-transfer components that we attribute to the inverse Faraday effect and optical spin-transfer torque, respectively. The inverse Faraday effect is approximately the same in Fe, Ni and Co, but the optical spin-transfer torque is strongly enhanced by adding a Pt capping layer. Our work provides quantitative data for testing theories of light–material interactions in metallic ferromagnets and multilayers.

## Abstract (web summary)

Optical switching of ferromagnets has attracted interest for use in ultrafast spintronics but the physical origin of the effect remains unclear. Here the authors determine the contributions of two proposed mechanisms, the inverse Faraday effect and optical spin-transfer torque.

Manipulation of magnetization via light is a key aspect of ultrafast spintronics. Beaurepaire *et al*.^1^ demonstrated that photon energy can be transferred to magnetization on a femtosecond time scale in a metallic ferromagnet. Later, Stanciu *et al*. showed that circular polarization of light can switch magnetization of a metallic ferrimagnet without the use of a magnetic field^2^. These results have led to the emerging field of all-optical helicity-dependent switching (AO-HDS)^2^,^3^,^4^,^5^,^6^,^7^. Until recently, AO-HDS was confined to ferrimagnetic systems, in which two sublattices are antiferromagnetically coupled; the mechanism of switching is connected to the compensation temperature, where the magnetizations of sublattices sum to zero^8^,^9^,^10^. Based on this understanding, however, the recent observation of AO-HDS in metallic ferromagnets was unexpected^11^. Helicity-dependent switching require a mechanism for angular momentum transfer from light to magnetization, but the mechanism for metallic ferromagnets is unknown.

The direct excitation of spin populations using light has been investigated primarily in semiconductors^12^,^13^,^14^,^15^,^16^,^17^. Circularly polarized light can generate spin-polarized electrons in the conduction band due to the optical selection rules for dipole transitions^12^,^13^,^14^,^15^,^16^,^17^; this mechanism, often referred to by the term ‘optical orientation', has practical importance for the generation of spin-polarized electron beams by photocathodes. In a semiconductor that has a net magnetic moment, spin-polarized electrons can interact with the magnetization of the semiconductor via optical-spin-transfer-torque (OSTT)^18^,^19^. OSTT in a semiconductor is the combination of two well-known mechanisms: optical orientation^12^,^13^,^14^,^15^,^16^,^17^ and spin-transfer torque between spin-polarized electrons and local magnetization^20^,^21^,^22^.

An additional mechanism for optical helicity-driven magnetization dynamics is the inverse Faraday effect (IFE). IFE was first discovered in insulating paramagnets^23^,^24^,^25^; IFE-driven magnetization dynamics has been reported in experiments on both insulating^26^,^27^ and metallic ferrimagnets^28^; recently, IFE was invoked to explain terahertz-frequency emission by metallic ferromagnets^29^. Although IFE has been proposed as the mechanism for AO-HDS for ferromagnetic metals^11^, a rigorous theory for IFE in metals is still under development^30^,^31^,^32^,^33^,^34^,^35^,^36^. The original theory of refs 24, 25 developed for insulators explains IFE in terms of an interaction Hamiltonian that couples angular momentum of light and material; this interaction Hamiltonian induces an optomagnetic field which is proportional to |*E*|^2^, where *E* is the electric field of light inside the material. This optomagnetic field produces a field-like torque that drives magnetization dynamics.

Theories of IFE are sometimes based on induced magnetization rather than an optomagnetic field^32^,^34^. To clarify the discussion, we note key differences between the IFE-driven and OSTT-driven magnetization. First, the IFE-induced magnetization is derived from a second-order perturbation with respect to *E*-field of light^32^,^34^, while the OSTT-driven magnetization is derived from a first-order perturbation^14^,^17^. Second, the relationship between magnetization, *m*, and light intensity, *I*, is different: *I*∝*m* for IFE; 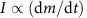 for OSTT. In other words, the IFE-induced magnetization is an equilibrium quantity calculated from the second-order density matrix response^32^,^34^, while the OSTT-induced magnetization is derived from the rate of spin generation, calculated from probability of interband transitions^14^,^17^. The IFE-induced *B*-field and magnetization can be related by *m*=*χ*_m_*B*, where *χ*_m_ is the static magnetic susceptibility^32^. However, for the IFE-driven magnetization by short optical pulse, we must consider dynamic behaviour. For example, the alignment of magnetization along the *B*-field will take a few nanoseconds for Co as magnetization undergoes a damped precessional motion to approach the equilibrium position. However, if the alignment of magnetization occurs during the pulse duration, we can treat IFE as a magnetization rather than a *B*-field^32^,^34^. In our analysis, we assume that the timescale for IFE to induce magnetization is much longer than the pulse duration, and treat IFE as a transient *B*-field created by the optical pulse and solve the torque equation.

Our experiments are designed to understand the magnitude and mechanisms of the optical-helicity-driven magnetization dynamics in metallic ferromagnets (FM): Co, Fe and Ni. We observe that circular polarized light produces magnetization dynamics that is explained by a combination of a spin-transfer torque that we attribute to spin polarization generated by OSTT and a field-like torque that we attribute to optomagnetic field generated by IFE. With a thin Au or MgO capping layer on top of the FM layer, the field-like torque dominates the magnetization dynamics. Replacing the Au or MgO capping layer with a Pt capping layer results in a significant enhancement of the spin-transfer torque, while the field-like torque remains approximately constant.

## Results

### Sample preparation and optical measurement

The film structure that we study is sapphire substrate/FM(10)/capping(*x*), where FM is Co, Fe or Ni with thickness of 10 nm, and capping(*x*) is Au, MgO or Pt with thickness of *x* nm. All layers are deposited by magnetron sputtering with base pressure of <5 × 10^−8^ Torr. For optical measurements, we use a time-resolved pump-probe technique (see Methods). The circularly polarized pump light is incident on the substrate side of the samples. A photon with left circular polarization (LCP) and right circular polarization (RCP) carries a spin angular momentum of +*ħ* and −*ħ*, respectively. (For light helicity, we adapt the view point from the receiver, which is the most common convention in optics^37^.) The linearly polarized probe light is incident on the surface side of the samples and detects the magnetization dynamics by magneto-optical Kerr effect (MOKE). We use high frequency modulation and balanced detection to minimize noise level (see Methods). All experiments are performed at room temperature.

### Two orthogonal torques by optical pulse

**Figure 1:**
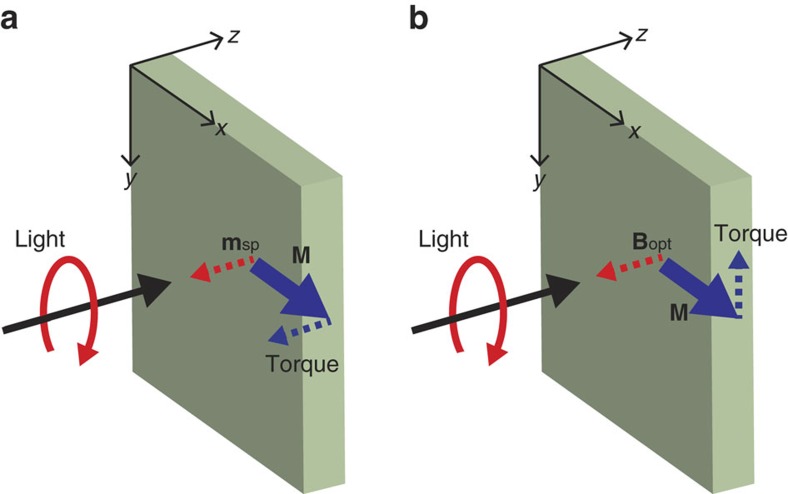
Schematic representation of two orthogonal torques. Circularly polarized light, incident on magnetization of ferromagnet (**M**), generates either spin polarization (**m**_sp_) via optical orientation or optomagnetic field (**B**_opt_) via inverse Faraday effect. (Black arrow indicates a wavevector of light along the *z*-direction, and red circular arrow indicates left-circular polarization.) (**a**) The **m**_sp_ (red dotted arrow) rotates **M** (blue solid arrow) to the *z*-direction by spin-transfer torque (blue dotted arrow). (**b**) The **B**_opt_ (red dotted arrow) rotates **M** (blue solid arrow) to the *y*-direction by field-like torque (blue dotted arrow).

Figure 1 illustrates our assignments of two orthogonal torques to helicity-driven magnetization dynamics: the spin-transfer torque by OSTT and the field-like torque by IFE. Circularly-polarized pump light is incident on a FM thin film in the *z*-direction; the magnetization of FM lies in the *x*-direction. The magnetization dynamics driven by OSTT and IFE during the pump pulse can be expressed as^18^,^20^,


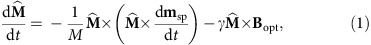


where 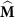 is the unit vector of magnetization of FM, *M* is the magnitude of magnetization of FM, **m**_sp_ is the OSTT-driven spin polarization, *γ* is the gyromagnetic ratio and **B**_opt_ is the IFE-driven optomagnetic field. The **m**_sp_ applies torque to the *z*-direction, while the **B**_opt_ applies torque to the *y*-direction. When magnetization is tilted from the equilibrium position by transient torque during the optical pulse, its dynamics after the optical pulse is governed by the effective *B*-field, determined by shape anisotropy, crystalline anisotropy and external magnetic field. Since the effective *B*-field is along the *x*-direction, the magnetization dynamics is a damped precessional motion in the *y–z* plane with the centre axis in the *x*-direction.

### Time-resolved MOKE measurement

**Figure 2:**
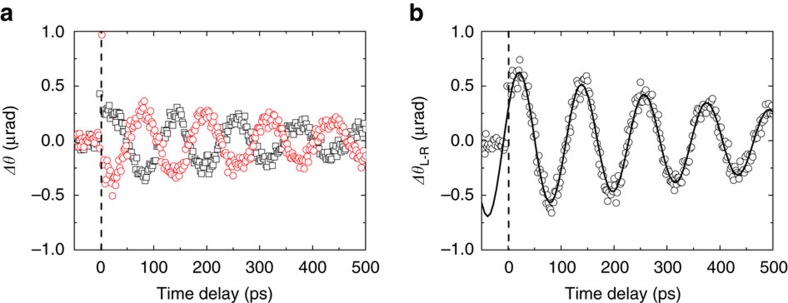
Polar MOKE result of the Co(10)/Au(2) sample. (**a**) Data with left circularly polarized (LCP) (black squares) and right circularly polarized (RCP) (red circles) pump. (**b**) The helicity-dependent magnetization dynamics obtained by subtraction of the data with LCP and RCP. The black line is the damped cosine function of *A*cos(2π*ft−φ*)exp(−*t/τ*), where *A*=0.65 μrad, *φ*=65° and *τ*=600 ps. The dashed vertical line indicates time delay of 1 ps. All measurements are done with incident pump fluence of 10 J m^−2^.

We measure the *z*-component of magnetization (*M*_*z*_) dynamics of the Co(10)/Au(2) sample using polar MOKE. Pump light triggers precession of magnetization, and this magnetization dynamics changes sign with pump helicity (Fig. 2a). A small helicity-independent (asymmetric) component of the magnetization dynamics is due to a small misalignment between the orientations of the crystalline anisotropy and the applied magnetic field (Supplementary Note 1). We extract the helicity-dependent (symmetric) component by taking the difference (Δ*θ*_L−R_) between LCP and RCP (Fig. 2b). We fit the data for Δ*θ*_L−R_ with a damped cosine function of the form cos(2π*ft*−*φ*)exp(−*t*/*τ*) where *f* is the precession frequency, *f*=8.5 GHz, *t* is the time delay between pump and probe, *φ*=65° is the phase delay and *τ*=600 ps is the time constant for exponential decay. Later, we convert Δ*θ*_L−R_ to the relative change of magnetization (Δ*M*/*M*) using 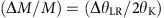, where *θ*_K_ is the Kerr rotation angle corresponding to saturation magnetization (see Table 1). The parameters *f* and *τ* are determined by saturation magnetization and damping constant of FM, respectively (Supplementary Note 2).

**Figure 3:**
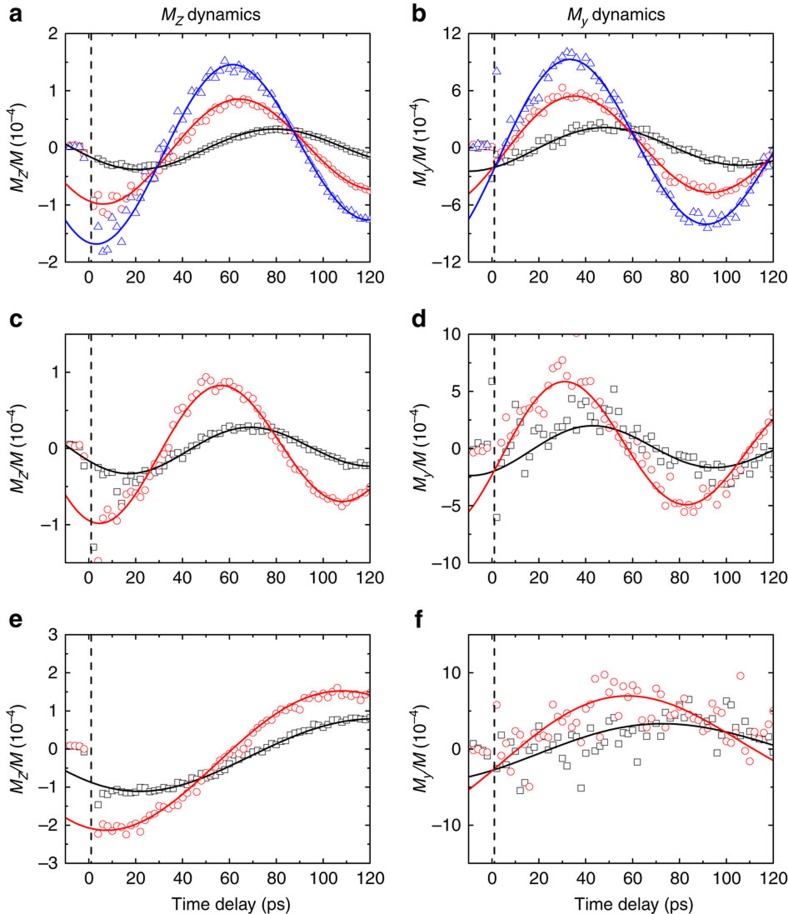
Helicity-driven *M*_*z*_ and *M*_*y*_ dynamics of all samples. The different sample capping layers are Au(2) (black squares), Pt(2) (red circles) and Pt(4) (blue triangles). (**a**,**b**) Data for Co(10)/capping. (**c**,**d**) Data for Fe(10)/capping. (**e**,**f**) Data for Ni(10)/capping.

The phase delay, *φ*, of *M*_*z*_ dynamics is determined by the direction of the initial tilting of magnetization. When the initial tilting of magnetization is along the *z*-direction, *φ* should be 0° (Fig. 2 of ref. 18). When the initial tilting of magnetization is along the *y*-direction, *φ* should be 90° (Fig. 2 of ref. 27). The large *φ* of 65° suggests that the initial tilting of magnetization is closer to the *y*-direction than *z*-direction. We also observe a large phase delay in the *M*_*z*_ dynamics in Fe and Ni, (Fig. 3), and when a different capping layer, MgO, is used (Supplementary Note 3).

To obtain a more complete picture of the magnetization dynamics, we measure the *y*-component of magnetization (*M*_*y*_) dynamics using longitudinal MOKE in the *y–z* plane (Fig. 3). *M*_*y*_ dynamics is 90° out of phase with *M*_*z*_ dynamics and has 4∼6 times larger amplitude than *M*_*z*_ dynamics due to the shape anisotropy of FM. We also measure the *x*-component of magnetization dynamics using longitudinal MOKE in the *x–z* plane, but found no helicity dependence. Although energy of light induces ultrafast demagnetization in the *x*-direction, there is no optical-helicity dependence on *M*_*x*_ dynamics (Supplementary Note 4).

### Effect of a Pt capping layer

Replacing the Au capping layer with a Pt capping layer decreases phase delay and increases the amplitude of *M*_*z*_ dynamics (Fig. 3). Especially with Co(10)/Pt(4) samples, the amplitude of precession is about four times larger than that of Co(10)/Au(2) sample, and the phase delay decreases to ∼10°. From optical calculation, the Pt layer absorbs 18% and 30% of light energy absorbed in the Co(10)/Pt(2) and Co(10)/Pt(4) structure, respectively; the Au layer absorbs 1% of light energy absorbed in the Co(10)/Au(2) structure (Supplementary Note 5).

**Figure 4:**
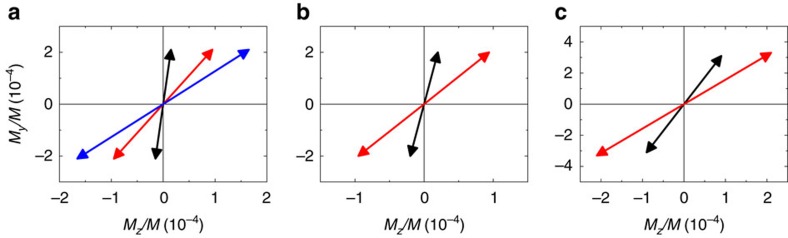
Helicity-driven *M*_*z*_ and *M*_*y*_ tilting at *t*=1 ps. (**a**) Determination of Co(10)/capping. (**b**) Determination of Fe(10)/capping. (**c**) Determination of Ni(10)/capping. Capping layers are Au(2) (black arrows), Pt(2) (red arrows) and Pt(4) (blue arrows). Arrows with negative/positive *M*_*z*_ and *M*_*y*_ are for left/right circularly polarized pumping.

We determine the initial *M*_*z*_ and *M*_*y*_ tilting immediately after the pump pulse (*t*=1 ps) by analysing the amplitude and phase of *M*_*z*_ and *M*_*y*_ dynamics shown in Fig. 3. The initial *M*_*y*_/*M* tilting is insensitive to the capping layers: −2 × 10^−4^ for Co and Fe and −3 × 10^−4^ for Ni samples with LCP pump (the sign changes with RCP). However, the initial *M*_*z*_/*M* tilting is highly sensitive to the capping layers: the initial *M*_*z*_/*M* tilting increases from −0.2 × 10^−4^ for Co(10)/Au(2) sample to −1.7 × 10^−4^ for Co(10)/Pt(4) sample with LCP pump (the sign changes with RCP). The similar enhancement of the *M*_*z*_/*M* tilting is also observed in Fe and Ni samples (Fig. 4).

### Quantification of IFE

From the initial *M*_*y*_, we evaluate the optomagnetic field needed to generate the field-like torque. The magnetization tilting along the *y*-direction generated by the pump pulse is related to the optomagnetic field by


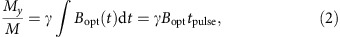


where *B*_opt_ is the optomagnetic field averaged over pulse duration, *t*_pulse_=1.1 ps is the pulse duration of pump. *M*_*y*_/*M*=−2 × 10^−4^ corresponds to *B*_opt_=1 mT along the negative *z*-direction. Considering the |*E*|^2^≈10^15^ V^2^ m^−2^ inside FM (Supplementary Note 6), where *E* is the amplitude of electric field of light, *B*_opt_/|*E*|^2^≈10^−18^ T m^2^ V^−2^. This value of the optomagnetic field is close to the value derived from a study of an insulating ferrimagnet^27^ (Supplementary Note 7).

According to theory for transparent materials, the optomagnetic field generated by IFE can be related to the Faraday effect^38^. Faraday rotation (*θ*_F_) is due to the difference in the real part of refractive index (*n*_L/R_) of LCP and RCP and increases linearly with the path length (*l*) of light inside materials as 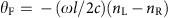, where *ω* is the angular frequency of light, and *c* is the speed of light in vacuum. (We adopt the sign convention from ref. 39.) Adopting the view point that helicity-dependent refractive indexes are a result of helicity-dependent resonant frequencies of electron (*ω*_L/R_) (ref. 38), the Hamiltonian for Faraday effect can be described as


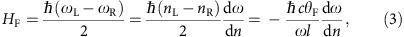


where *θ*_F_/*l* is 6.3 × 10^5^, 6.1 × 10^5^ and 1.7 × 10^5^ rad m^−1^ for Co, Fe and Ni, respectively at wavelength of 830 nm (ref. 40) (values at wavelength 784 nm have not been reported), 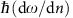 is −1 eV for Co and Ni and −2 eV for Fe at wavelength of 784 nm (ref. 41). If we assume that *H*_F_ is responsible for IFE as well, the IFE-driven *B*_opt_ can be expressed as 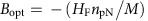, where *n*_pN_ is the photon density inside the material. The *n*_pN_ can be obtained from |*E*|^2^ by 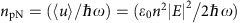, where 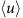 is the time-averaged energy density of light inside FM, and *ɛ*_0_ is the vacuum permittivity. Then the intensity-normalized *B*_opt_ is,


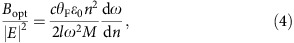


which is calculated to be 1 × 10^−18^ T m^2^ V^−2^ for Co and Ni and 2 × 10^−18^ T m^2^ V^−2^ for Fe along the negative *z*-direction for LCP. These estimates are consistent with our experimental results and suggest that the Hamiltonian responsible for Faraday effect is a useful approximation for IFE.

We compare our result to reported IFE theories for metals. Mondal and co-workers estimated *B*_opt_/|E|^2^≈(1+*χ*_e_)10^−22^ T m^2^ V^−2^, where *χ*_e_ is the electrical susceptibility^33^. Using the relation, *ɛ*_r_=1+*χ*_e_, where *ɛ*_r_ is the relative permittivity, and *ɛ*_r_ of −16.5+*i* 23.3 for Co at wavelength of 784 nm (ref. 41), and |*E*|^2^≈10^15^ V^2^ m^−2^ in our case, *B*_opt_≈3 × 10^−6^ T. The authors of ref. 33 raised a possibility of much larger *χ*_e_ for Fe relating *χ*_e_ to the anomalous Hall coefficient. Berritta and co-workers calculate the IFE-induced magnetization in metallic ferromagnets and related it to *B*_opt_ (ref. 34). Converting their calculation with the *I*_0_=10^14^ W m^−2^ to our case with *I*_0_≈10^13^ W m^−2^, *B*_opt_≈2∼40 T. Note that there is asymmetry for LCP and RCP because magnetization direction and light propagation direction is the same in their case while in our experiments the magnetization is orthogonal to the direction of light propagation. Qaiumzadeh and co-workers calculate *B*_opt_ in metallic ferromagnets from the direct optical transition of spin-split sub-bands^35^. Since magnetization and light propagation lie to the same direction, their results also show asymmetry for LCP and RCP. Converting their calculation with the *E*_0_=10^9^ V m^−1^ to our case with *E*_0_≈10^8^ V m^−1^, *B*_opt_≈0.02∼0.2 T. Freimuth and co-workers consider both IFE and OSTT effects on metallic ferromagnets^36^. They show that both IFE and OSTT depend on quasiparticle broadening (*Γ*) and spin–orbit interaction. With a *Γ*=25 meV for room temperature, they predict *B*_opt_ of 20 and 1.5 mT for Co and Fe, respectively, at *I*_0_≈10^13^ W m^−2^. The result for Fe is close to our experiment but not for Co.

### Quantification of OSTT

From the initial *M*_*z*_, we evaluate the spin polarization for OSTT. When the light-induced spin polarization is fully absorbed by magnetization of ferromagnet, the *M*_*z*_ tilting of ferromagnet by pump pulse is directly related to the spin polarization by,


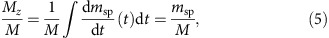


where *m*_sp_ is the spin polarization integrated over the pulse duration. From *M*_*z*_/*M*, the *m*_sp_ is 30, 130 and 230 A m^−1^ for Co(10)/Au(2), Co(10)/Pt(2) and Co(10)/Pt(4), respectively. Considering the light absorption in Pt (18% for Co(10)/Pt(2) and 30% for Co(10)/Pt(4)), we conclude that much larger *m*_sp_ is coming from Pt than from Co.

We use these data to determine the degree of spin polarization (DSP), a parameter often used in discussions of optical orientation in semiconductors. DSP is the ratio between spin polarized electrons (*n*_s_) and total electrons (*n*_tot_) excited by dipole transitions. Since *n*_s_ is related to *m*_sp_, and *n*_tot_ is related to the amount of light absorption, DSP can be derived from,


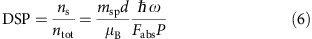


where *d*=10 nm is the thickness of the Co layer, *μ*_B_ is the Bohr magneton, *F*_abs_=6 J m^−2^ is the total absorbed fluence (determined from *F*_abs_=*F*_in_(1−*R*−*T*), where *F*_in_ is incident pump fluence of 10 J m^−2^, *R* is reflectance and *T* is transmittance), *P*=0.3 is the percentage of light absorption of Pt in the Co(10)/Pt(4), and *ħω*=1.58 eV is the photon energy. The difference of *m*_sp_ between Co(10)/Pt(4) and C(10)/Au(2) samples leads to DSP≈0.03 for Pt. (We estimate DSP of Co, Fe and Ni is at least one order of magnitude smaller than that of Pt.) Note that DSP of GaAs is 0.5 at a photon energy of 1.58 eV (ref. 14). The large DSP of GaAs is due to the large energy splitting in the valence band, between *P*_1/2_ and *P*_3/2_ bands, at the Gamma point. A theoretical calculation of DSP of transition metals will require consideration of the full band structure and energy splitting due to spin-orbit coupling.

The authors of ref. 36 theoretically calculate both IFE and OSTT contribution in metallic ferromagnets. They show that IFE can dominate over OSTT at least in Co, but they do not investigate non-magnetic metals, like Pt^36^. The authors of ref. 34 theoretically calculate the IFE-driven magnetization of several non-magnetic and ferromagnetic metals. They show that the IFE-driven magnetization is several times larger in Au and Pt than in Co, Fe and Ni due to larger spin-orbit coupling^34^.

In our analysis, light absorption by dipole transitions is the fundamental mechanism for OSTT but not for IFE (for IFE, light absorption is considered only to calculate the decay of *E*-field through sample thickness). However, recent theories for IFE predict that light absorption plays an important role to induce spin and orbital magnetization^34^,^36^. We argue that IFE should be treated as *B*-field for short optical pulse because the IFE-induced magnetization is an equilibrium property. However, if IFE can generate magnetization on the time scale of the pulse duration, IFE can contribute to *m*_sp_.

We compare the initial *M*_*z*_ with theoretical IFE-induced magnetization (*m*_IFE_) in Pt assuming timescale for *m*_IFE_ in Pt is shorter than the pulse duration. At *I*_0_=10^13^ W m^−2^ and *ħω*=1.58 eV, *m*_IFE_ in Pt are approximately 40 and 400 A m^−1^, respectively, for spin and orbital (sign is opposite for spin and orbital magnetization at given light helicity)^34^. When all spin and orbital *m*_IFE_ of Pt is transferred to Co magnetization (*m*_Co_), which is mostly spin magnetization, during the pulse duration, the initial *m*_Co_ along the *z*-direction can be related with *m*_IFE_ by, *m*_Co_*d*_Co_=*m*_IFE_*d*_Pt_, where *d*_Co_ and *d*_Pt_ are thickness of Co and Pt layers. Then *m*_Co_ is estimated to be 8 and 80 A m^−1^, respectively, by spin and orbital *m*_IFE_ of Pt with the Co(10)/Pt(2) sample, and it increases twice with the Co(10)/Pt(4) sample. The estimated *m*_Co_ by the orbital *m*_IFE_ of Pt is close to experimental observation. Note that this estimation is based on two assumptions: IFE can induce magnetization in Pt on a timescale of <1 ps; the orbital magnetization of Pt can be transferred to the spin magnetization of Co on a timescale of <1 ps. The orbital magnetization occurs during the dipole transition as well, but its effect on OSTT is often ignored for semiconductors^18^. Recent theory concludes that orbital magnetization has a negligible effect on magnetization dynamics of metallic ferromagnet^36^.

Another consideration for the *M*_*z*_ tiling is a spin relaxation. The light-induced spin polarization can relax to the environment before applying a torque on magnetization of FM when the spin relaxation time (*τ*_s_) is short enough. The time scale of *τ*_s_ can be estimated from spin relaxation length (*l*_s_) using 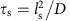, where *D* is electronic diffusivity. The reported *l*_s_ of Pt has a wide range 1∼10 nm, but it is related with electrical conductivity (*σ*)^42^. Considering *σ*=7 × 10^6^ Ω^−1^ m^−1^ of our Pt film, we estimate *l*_s_≈5 nm. With *l*_s_=5 nm and *D*=200 nm^2^ ps^−1^, obtained from *σ*, the spin relaxation time in Pt is *τ*_s_≈0.1 ps. The time scale for spin transfer torque (*τ*_stt_) in the Co/Pt bilayer can be estimated from 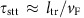, where *l*_tr_ is the travel length from Pt to Co, and *v*_F_ is the Fermi velocity of Pt. Considering *l*_tr_≈2 nm, *τ*_stt_ would be a few femtoseconds. When *τ*_stt_<<*τ*_s_, spin relaxation is not important. In addition, the spin relaxation should lead to saturation of *m*_sp_ with Pt thickness, but we do not see a saturation in the initial *M*_*z*_/*M* tilting up to Pt thickness of 4 nm.

### Ultrafast demagnetization

The light pulse not only changes the direction of magnetization but also reduces the magnitude of magnetization via ultrafast demagnetization. The peak demagnetization, |Δ*M*|/*M*, is 0.04, 0.04 and 0.25 for Co(10)/Pt(2), Fe(10)/Pt(2) and Ni(10)/Pt(2) samples, respectively (Supplementary Note 8). Ultrafast demagnetization is a result of energy transfer from light to magnetization and is related to the temperature excursion^1^,^43^,^44^. The temperature excursion per pulse is 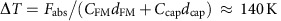, where *C*_FM_ and *C*_cap_ is heat capacity of FM and capping layer, respectively, and *d*_FM_ and *d*_cap_ is thickness of FM and capping layer, respectively. (Δ*T* of magnons can exceed 140 K during the pump pulse due to non-equilibrium between electrons, phonons and magnons^1^,^43^,^44^.) The more significant |Δ*M*|/*M* of Ni compared to Co or Fe is due to the relatively low Curie temperature of Ni^43^,^44^. We acknowledge that there are other theories for ultrafast demagnetization such as superdiffusive model^45^. According to the superdiffusive model, the larger demagnetization of Ni would be because spin dependence on electronic transport is more significant in Ni^45^. It has been proposed that IFE can lead to AO-HDS when the peak Δ*T* approaches the Curie temperature^11^. Recently, the possibility of AO-HDS by the combination of IFE and demagnetization was shown by simulation^46^. In this respect, a material with low Curie temperature is desirable for AO-HDS. The authors of refs 11, 46 did not, however, consider OSTT.

### Conclusions

We report the vectorial measurements of magnetization dynamics driven by helicity of light. We interpret the results in terms of two orthogonal torques: a field-like torque generated by an optomagnetic field (IFE); and a spin-transfer torque generated by a spin polarization (OSTT). We find that IFE dominates inside ferromagnetic layers, and OSTT mostly comes from a Pt capping layer. Despite our interpretation, it is possible that IFE causes a similar effect on Pt as OSTT does when the timescale for the IFE-induced magnetization is shorter than the pulse duration. Our findings present an important step towards understanding the coupling of angular momentum of light and magnetization. In particular, the capping layer dependence on OSTT and the material dependence on demagnetization can be useful in the design for materials for AO-HDS in metallic ferromagnets.

## Methods

### Pump-probe measurement

The centre wavelength of pump and probe is 784 nm. The full-width-at-half-maximum (FWHM) of time-correlation of pump and probe is 1.15 ps, which is mostly due to FWHM of pump as it gets broaden by the large dispersion of the electro-optic modulator: we estimate FWHM of 1.1 and 0.2 ps for pump and probe, respectively. The zero time delay (*t*=0 ps) is set to the centre of pump pulse.

### Noise suppression

We suppress noise level using synchronous detection using a high modulation frequency (10 MHz) combined with balanced detection. In our set-up, the noise level for polar MOKE detection is on the order of 0.1 μrad per 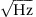 which corresponds to a fractional change of magnetization of about 10^−5^ for Co. We can further reduce noise by averaging multiple measurements.

### Data availability

The data that support the findings of this study are available from the corresponding author on request.

## Additional information

**How to cite this article:** Choi, G.-M. *et al*. Optical-helicity-driven magnetization dynamics in metallic ferromagnets. *Nat. Commun.* **8,** 15085 doi: 10.1038/ncomms15085 (2017).

**Publisher's note:** Springer Nature remains neutral with regard to jurisdictional claims in published maps and institutional affiliations.

## Supplementary Material

Supplementary Information Supplementary Figures, Supplementary Notes, Supplementary Table and Supplementary References

Peer Review File

## Figures and Tables

**Table 1:** Amplitude and phase of *M*_*z*_ precession of all samples.

	**Co(10)/Au(2)**	**Co(10)/Pt(2)**	**Co(10)/Pt(4)**	**Fe(10)/Au(2)**	**Fe(10)/Pt(2)**	**Ni(10)/Au(2)**	**Ni(10)/Pt(2)**
Δ*θ*_L−R_ (μrad)	0.7±0.2	1.6±0.3	2.3±0.5	0.5±0.1	1.3±0.3	0.8±0.2	1.4±0.3
*θ*_K_ (mrad)	−8.0±0.8	−7.8±0.8	−6.9±0.7	−6.5±0.7	−6.5±0.7	−3.3±0.3	−3.2±0.3
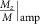 (10^−4^)	−0.4±0.1	−1.0±0.3	−1.7±0.5	−0.4±0.1	−1.0±0.3	−1.2±0.4	−2.2±0.6
*φ* (°)	65±5	22±5	12±5	63±5	19±5	45±5	20±5

The Δ*θ*_L−R_ is the peak Kerr rotation due to the optical-helicity-dependent part of the magnetization dynamics. The *θ*_K_ is the static Kerr rotation due to full magnetization. The 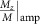 and *φ* are amplitude and phase of *M*_*z*_ precession, respectively.

## References

[b1] Beaurepaire E., Merle J.-C., Daunois A. & Bigot J.-Y. Ultrafast spin dynamics in ferromagnetic nickel. Phys. Rev. Lett. 76, 4250–4253 (1996). 10061239 10.1103/PhysRevLett.76.4250

[b2] Stanciu C. D. . All-optical magnetic recording with circularly polarized light. Phys. Rev. Lett. 99, 047601 (2007). 17678404 10.1103/PhysRevLett.99.047601

[b3] Khorsand A. R. . Role of magnetic circular dichroism in all-optical magnetic recording. Phys. Rev. Lett. 108, 127205 (2012). 22540622 10.1103/PhysRevLett.108.127205

[b4] Alebrand S., Hassdenteufel A., Steil D., Cinchetti M. & Aeschlimann M. Interplay of heating and helicity in all-optical magnetization switching. Phys. Rev. B 85, 092401 (2012).

[b5] Alebrand S. . Light-induced magnetization reversal of high-anisotropy TbCo alloy films. Appl. Phys. Lett. 101, 162408 (2012).

[b6] Hassdenteufel A. . Thermally assisted all-optical helicity dependent magnetic switching in amorphous Fe_100−x_Tb_x_ Alloy films. Adv. Mater. 25, 3122–3128 (2013). 23616209 10.1002/adma.201300176

[b7] Kirilyuk A., Kimel A. V. & Rasing T. H. Laser-induced magnetization dynamics and reversal in ferrimagnetic alloys. Rep. Prog. Phys. 76, 026501 (2013). 23377279 10.1088/0034-4885/76/2/026501

[b8] Radu I. . Transient ferromagnetic-like state mediating ultrafast reversal of antiferromagnetically coupled spins. Nature 472, 205–208 (2011). 21451521 10.1038/nature09901

[b9] Ostler T. A. . Ultrafast heating as a sufficient stimulus for magnetization reversal in a ferrimagnet. Nat. Commun. 3, 666 (2012). 22314362 10.1038/ncomms1666

[b10] Mangin S. . Engineering materials for all-optical helicity-dependent magnetic switching. Nat. Mater. 13, 286–292 (2014). 24531398 10.1038/nmat3864

[b11] Lambert C.-H. . All-optical control of ferromagnetic thin films and nanostructures. Science 345, 1337–1340 (2014). 25147280 10.1126/science.1253493

[b12] Lampel G. Nuclear dynamic polarization by optical electronic saturation and optical pumping in semiconductors. Phys. Rev. Lett. 20, 491–493 (1968).

[b13] Pierce D. P. & Meier F. Photoemission of spin-polarized electrons from GaAs. Phys. Rev. B 13, 5484–5500 (1976).

[b14] Meier F. & Zakharchnya B. P. Optical Orientation North-Holland (1984).

[b15] Awschalom D. D., Warnock J. & von Molnár S. Low-temperature magnetic spectroscopy of a dilute magnetic semiconductor. Phys. Rev. Lett. 58, 812–815 (1887). 10.1103/PhysRevLett.58.812 10035043

[b16] Oiwa A., Mitsumori Y., Moriya R., Słupinski T. & Munekata H. Effect of optical spin injection on ferromagnetically coupled Mn apins in the III-V magnetic alloy semiconductor (Ga, Mn)As. Phys. Rev. Lett. 88, 137202 (2002). 11955121 10.1103/PhysRevLett.88.137202

[b17] Nastos F., Rioux J., Strimas-Mackey M., Mendoza B. S. & Sipe J. E. Full band structure LDS and k·p calculations of optical spin-injection. Phys. Rev. B 76, 205113 (2007).

[b18] Némec P. . Experimental observation of the optical spin transfer torque. Nat. Phys. 8, 411–415 (2012).

[b19] Ramsay A. J. . Optical spin-transfer-torque-driven domain-wall motion in a ferromagnetic semiconductor. Phys. Rev. Lett. 114, 067202 (2015). 25723242 10.1103/PhysRevLett.114.067202

[b20] Slonczewski J. C. Current-driven excitation of magnetic multilayers. J. Magn. Magn. Mater. 159, L1–L7 (1996).

[b21] Berger L. Emission of spin waves by a magnetic multilayer traversed by a current. Phys. Rev. B 54, 9353–9358 (1996). 10.1103/physrevb.54.9353 9984672

[b22] Stiles M. D. & Zangwill A. Anatomy of spin-transfer torque. Phys. Rev. B 66, 014407 (2002).

[b23] Pitaevskii L. P. Electric forces in a transparent dispersive medium. Sov. Phys. JETP 12, 1008–1013 (1961).

[b24] van der Ziel J. P., Pershan P. S. & Malmstrom L. D. Optically-induced magnetization resulting from the inverse Faraday effect. Phys. Rev. Lett. 15, 190–193 (1965).

[b25] Pershan P. S., van der Ziel J. P. & Malmstrom L. D. Theoretical discussion of the inverse Faraday effect, Raman Scattering, and related phenomena. Phys. Rev. 143, 574–583 (1966).

[b26] Kimel A. V. . Ultrafast non-thermal control of magnetization by instantaneous photomagnetic pulses. Nature 435, 655–657 (2005). 15917826 10.1038/nature03564

[b27] Hansteen F., Kimel A., Kirilyuk A. & Rasing T. H. Femtosecond photomagnetic switching of spins in ferrimagnetic garnet films. Phys. Rev. Lett. 95, 047402 (2005). 16090839 10.1103/PhysRevLett.95.047402

[b28] Stanciu C. D. . Ultrafast interaction of the angular momentum of photons with spins in the metallic amorphous alloy GdFeCo. Phys. Rev. Lett. 98, 207401 (2007). 17677737 10.1103/PhysRevLett.98.207401

[b29] Huisman T. J. . Femtosecond control of electric currents in metallic ferromagnetic heterostructures. Nat. Nanotech. 11, 455–458 (2016). 10.1038/nnano.2015.331 26854566

[b30] Popova D., Bringer A. & Blügel S. Theoretical investigation of the inverse Faraday effect via a stimulated Raman scattering process. Phys. Rev. B 85, 094419 (2012).

[b31] Raeliarijaona A., Singh S., Fu H. & Bellaiche L. Predicted coupling of the electromagnetic angular momentum density with magnetic moments. Phys. Rev. Lett. 110, 137205 (2013). 23581367 10.1103/PhysRevLett.110.137205

[b32] Battiato M., Barbalinardo G. & Oppeneer P. M. Quantum theory of the inverse Faraday effect. Phys. Rev. B 89, 014413 (2014).

[b33] Mondal R. . Relativistic interaction Hamiltonian coupling the angular momentum of light and the electron spin. Phys. Rev. B 92, 100402(R) (2015).

[b34] Berrita M., Mondal R., Carva K. & Oppeneer M. *Ab Initio* theory of coherent laser-induced magnetization in metals. Phys. Rev. Lett. 117, 137203 (2016). 27715112 10.1103/PhysRevLett.117.137203

[b35] Qaiumzadeh A. & Titov M. Theory of light-induced effective magnetic field in Rashba ferromagnets. Phys. Rev. B 94, 014425 (2016).

[b36] Freimuth F., Blügel S. & Mokrousov Y. Laser-induced torques in metallic ferromagnets. Phys. Rev. B 94, 144432 (2016).

[b37] Landi Degl'innocenti E. & Landolfi M. Polarization in Spectral Lines Kluwer Academic Publishers (2004).

[b38] Zvezdin A. K. & Kotov V. A. Modern Magnetooptics and Magnetooptical Materials Taylor & Francis Group (1997).

[b39] Atkinson R. & Lissberger P. H. Sign conventions in magneto-optical calculations and measurements. Appl. Opt. 31, 6076–6081 (1992). 20733810 10.1364/AO.31.006076

[b40] Coey J. M. D. Magnetism and Magnetic Materials Cambridge University Press (2009).

[b41] Johnson P. B. & Christy R. W. Optical constants of transition metals: Ti, V, Cr, Mn, Fe, Co, Ni, and Pd. Phys. Rev. B 9, 5056–5070 (1974).

[b42] Sagasta E. . Tuning the spin Hall effect of Pt from the moderately dirty to the superclean regime. Phys. Rev. B 94, 060412(R) (2016).

[b43] Koopmans B. . Explaining the paradoxical diversity of ultrafast laser-induced demagnetization. Nat. Mater. 9, 259–265 (2010). 20010830 10.1038/nmat2593

[b44] Choi G.-M., Moon C.-H., Min B.-C., Lee K.-J. & Cahill D. G. Thermal spin-transfer torque driven by the spin-dependent Seebeck effect in metallic spin-valves. Nat. Phys. 11, 576–581 (2015).

[b45] Battiato M., Carva K. & Oppeneer P. M. Superdiffusive spin transport as a mechanism of ultrafast demagnetization. Phys. Rev. Lett. 105, 027203 (2010). 20867735 10.1103/PhysRevLett.105.027203

[b46] Cornelissen T. D., Córdoba R. & Koopmans B. Microscopic model for all optical switching in ferromagnets. Appl. Phys. Lett. 108, 142405 (2016).

