# Supplementary material for: Optical-helicity-driven magnetization dynamics in metallic ferromagnets
Source: Nat Commun. 2017 Apr 18;8:15085. doi: 10.1038/ncomms15085 (PMC5399298; doi:10.1038/ncomms15085)
Supplement: Supplementary Information — Supplementary Figures, Supplementary Notes, Supplementary Table and Supplementary References [file ncomms15085-s1.pdf]

Supplementary Note 1: Extracting helicity-dependent part

The magnetization dynamics depends on the optical helicity and the alignment between the crystalline anisotropy and applied magnetic field (Supplementary Figure 1). The easy axis of the crystalline anisotropy lies in the x-y plane and makes an angle, ξ, with the x-axis of the applied magnetic field of 0.05 T. The pump pulse rapidly heats the magnetization which leads to an abrupt change of magnetization anisotropy at near zero time delay. When ξ is not zero, this rapid heating creates an abrupt change in the anisotropy field that triggers a coherent precession of the magnetization [1]. When $\phi \approx 0\,^{\mathrm{o}}$, the rapid heating does not create a pulse in the anisotropy field, and the magnetization dynamics is symmetric with optical helicity.

We extract the helicity-dependent part of the magnetization dynamics by plotting the difference between LCP and RCP ($\Delta\theta_{\mathrm{L\text{-}R}}$). Note that the helicity-dependent component of the magnetization dynamics is independent of ξ (Supplementary Figure 1 d).

Supplementary Note 2: Precession frequency and damping constant

We determine the precession frequency and damping constant of all samples from M_z precession up to 500 ps (Supplementary Figure 2). The precession frequency is in good agreement with the prediction of Kittel's equation, $f = \frac{\gamma_{\mathrm{e}}}{2\pi}\sqrt{B_x(B_x + \mu_0 M_{\mathrm{S}})}$, where γ_{e} = 1.76×10^{11} rad s^{-1} T^{-1} is the electron gyromagnetic ratio, B_x = 0.05 T is the in-plane magnetic field, μ_0 is the vacuum permeability, and M_{S} is the saturation magnetization of Co of 1.4×10^6 A m^{-1}. The precession frequencies of Fe and Ni samples are different than for Co due to the larger and smaller saturation magnetization: M_{S} = 1.7×10^6 and 0.5×10^6 A m^{-1} for Fe and Ni, respectively. The precession

frequency does not depend on capping materials, but the damping constant does: Pt capping results in a slightly higher damping than Au capping. The precession frequency and damping constant are summarized in the Supplementary Table 1.

Supplementary Note 3: Comparison between MgO capping and Au capping

In addition to Pt and Au capping, we measured optical-helicity-dependent magnetization dynamics of Co with MgO capping (Supplementary Figure 3). A 5 nm-thick MgO layer was deposited on top of a 10 nm-thick Co layer in the same deposition chamber, i.e., without breaking vacuum. An additional 1 nm-thick Ta layer was deposited on top of the MgO layer to ensure passivation of MgO. The *z*-component of magnetization dynamics of the Co(10)/MgO(5) sample shows a phase delay, which is close to the phase delay of the Co(10)/Au(2) sample. Since the MgO layer does not absorb light, it does not have OSTT. A similar phase delay between Co(10)/MgO(5) and Co(10)/Au(2) samples implies that the Au capping layer does not contribute significantly to OSTT. One of reasons for this result is that the amount of light absorption in Au is much smaller than in Co (see Supplementary Note 5).

Supplementary Note 4: M_x dynamics

We measure M_x dynamics using longitudinal MOKE in *x*-*z* plane with incidence angle of about 10 degree. The Kerr rotation in this geometry has contributions both from M_z and M_x. M_z contribution can be subtracted because it does not change sign with the sign change of external magnetic field, which is along the *x*-direction, while M_x contribution does. Both LCP and RCP

pump produces ultrafast demagnetization of ferromagnet, which lies to the *x*-direction, but there is no difference between LCP and RCP (Supplementary Figure 4). It is known that ultrafast demagnetization can generate spins along the magnetization direction, *x*-direction in our case [2, 3]. This spin can travel through the capping layer, come back to ferromagnet, applies torque on magnetization along the *x*-direction. If there is any helicity-dependent effect on this process, this spin current should lead to helicity-dependent torque in the *x*-direction. However, we do not see any helicity dependence in M_x dynamics. In our experiments, we align magnetization and light propagation directions orthogonal to each other. When magnetization and light propagation directions are parallel, spin-polarization from ultrafast demagnetization would dominate, and spin polarization from optical helicity will be overwhelmed. However, when magnetization and light propagation directions are orthogonal, spin polarization from optical helicity can be distinguished from spin polarization from ultrafast demagnetization.

Supplementary Note 5: Calculation of light absorption

We calculate the Poynting vector of the pump light as it passes through the sapphire/ Co (10 nm)/ Pt or Au (2 nm) structure using a transfer matrix method. Refractive indices of 1.76 for sapphire, 2.5 + *i* 4.7 for Co, 0.2 + *i* 4.6 for Au, and 2.8 + *i* 4.9 for Pt, are taken from literature values [4, 5] (Supplementary Figure 5). The decrease of the Poynting vector represents the light absorption. For the Co(10)/Au(2) structure, the total light absorption, (1-*R*-*T*), where *R* is reflectance and *T* is transmittance, is 0.55, where the 10 nm-thick Co layer absorbs 99 % of the total light absorption and the 2 nm-thick Au layer absorbs 1 %. For the Co(10)/Pt(2) structure, the total light absorption is 0.58, where the 10 nm-thick Co layer absorbs 82 % of the total and the 2

nm-thick Pt layer absorbs 18 %. For the Co(10)/Pt(4) structure, the total light absorption is 0.57, where the 10 nm-thick Co layer absorbs 70 % of the total and the 4 nm-thick Pt layer absorbs 30 %.

Supplementary Note 6: Calculation of electric field

We calculate the electric field of the pump light inside Co of the sapphire/ Co (10 nm)/ Au or Pt (2 nm) structure (Supplementary Figure 6). The electric field of light is related to intensity, I, by $I = \frac{1}{2} c\varepsilon E_0^2$, where c is the speed of light, ε is the permittivity, and E_0 is the amplitude of electric field of light. The initial intensity of the pump right before the sample is $\approx 10^{13}$ W m^{-2} from $I_{\mathrm{in}} = \frac{F_{\mathrm{in}}}{\Delta t_{\mathrm{pulse}}}$, where F_{in} is the incident fluence of the pump of 10 J m^{-2} and $\Delta t_{\mathrm{pulse}}$ is the width of the pump of 1.1 ps. (Although the pump is a Gaussian function of time, we use the time-averaged intensity for simplicity). The initial intensity of electric field before the sample is then E_0^2 = 7.5×10^{15} V^2 m^{-2}. From this initial E_0^2, we calculate $|E|^2$ inside Co using a transfer matrix method with the same refractive indexes as in Supplementary Note 5. From the transfer matrix method we obtain two components of electric field of light inside Co: the $+z$-propagating E_+, which we refer to as transmitted light, and $-z$-propagating E_-, which we refer to as reflected light. This reflected light reverses not only the propagating direction but also handedness of circular polarization. However, in terms of the optomagnetic field, B_{opt}, sign changes in the propagation direction and handedness of polarization cancel each other; thus, the sign of B_{opt} of the reflected light is the same as that of the transmitted light. Therefore, the relevant component of the electric field inside Co for B_{opt} is the sum of $|E_+|^2$ and $|E_-|^2$. The sum of $|E_+|^2$ and $|E_-|^2$ decreases through Co thickness. This sum averaged over thickness of the Co layer is 1.2×10^{15} V^2 m^{-2} and 1.1×10^{15} V^2 m^{-2} for the

Co(10)/Au(2) and Co(10)/Pt(2) structures, respectively. With the B_{opt} of 1 mT of the main text, $B_{opt}/|E|^2 \approx 10^{-18}$ T m^2 V^{-2}.

Supplementary Note 7: Comparison with insulating ferrimagnet

We compare our results for IFE to studies of an insulating ferrimagnet. The authors of Supplementary Ref. [6] provided an estimate for B_{opt} from the analysis of experiments of helicity-driven magnetization dynamics of an insulating ferrimagnet of $Lu_{1.69}Y_{0.65}Bi_{0.66}Fe_{3.85}Ga_{1.15}O_{12}$: B_{opt} = 0.6 T with incident pump intensity of $I = 10^{15}$ W m^{-2} and pulse width of Δt_{pulse}=0.1 ps. From $I = \frac{1}{2} c\varepsilon E_0^2$, the initial intensity of electric field before the sample is then E_0^2=7.5×10^{17} V^2/m^2. The electric field inside $Lu_{1.69}Y_{0.65}Bi_{0.66}Fe_{3.85}Ga_{1.15}O_{12}$ can be calculated from $E = \frac{2n_0}{n_0+n_1} E_0$, where n_0 is the refractive index of air and n_1 is the refractive index of $Lu_{1.69}Y_{0.65}Bi_{0.66}Fe_{3.85}Ga_{1.15}O_{12}$. Using the refractive index of typical garnet system of 1.8, E^2 is 3.8×10^{17} V^2 m^{-2}, and $B_{opt}/|E|^2 \approx 1.6\times10^{-18}$ T m^2 V^{-2}, comparable to our results for metallic ferromagnets.

Supplementary Note 8: Ultrafast demagnetization

We measure demagnetization of FM of FM(10)/Pt(2) samples by partially aligning magnetization into the z-direction with a perpendicular magnetic field of 0.45 T. Pump and probe are both linearly polarized and incident on the substrate side of samples. At the same incident fluence of 10 J m^{-2}, the peak of normalized demagnetization, $|\Delta M|/M$, is 0.04, 0.04, and 0.25 for Co, Fe, and Ni respectively. Previously, we have shown that demagnetization is determined by the changes in magnon temperature and Curie temperature of FM [7]. Since the temperature rise per

pulse, ~140 K, is similar for Co, Fe, and Ni, the large difference in $|\Delta M|/M$ is due to the difference in Curie temperature: Curie temperature is 1388, 1042, 627 K for Co, Fe, and Ni, respectively.

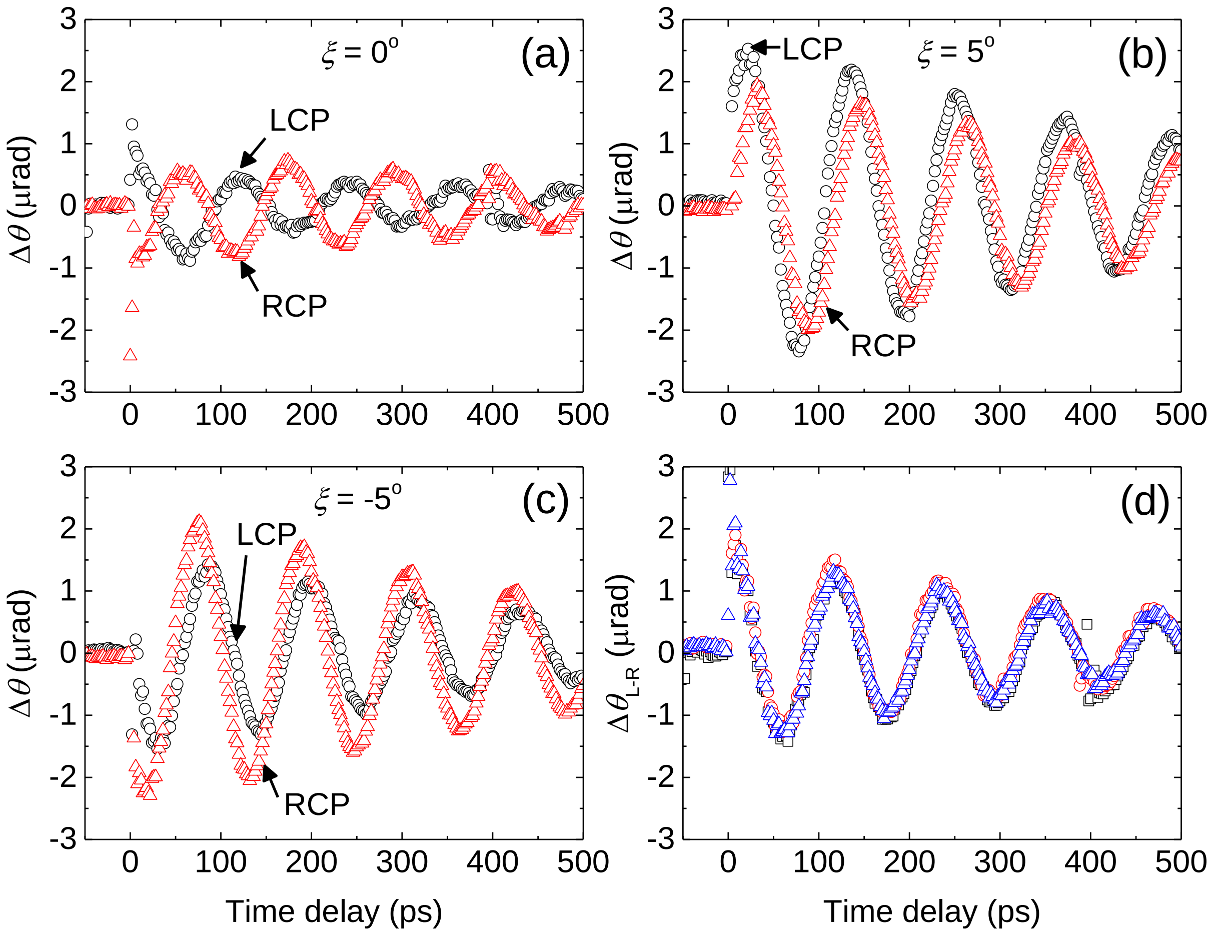

Supplementary Figure 1: MOKE results with different angle between crystalline anisotropy and external magnetic field Data with left circular polarized (LCP) and right circular polarized (RCP) pump and with the angle, ξ, between crystalline anisotropy and external magnetic field is controlled to 0 ^{o} (a), 5 ^{o} (b), and -5 ^{o} (c). (d) The helicity-dependent part is obtained from subtraction of the data collected with LCP and RCP pump of (a), (b), and (c). The black squares, red circles, and blue triangles are results with different ξ of 0 ^{o}, +5 ^{o}, and -5 ^{o}, respectively.

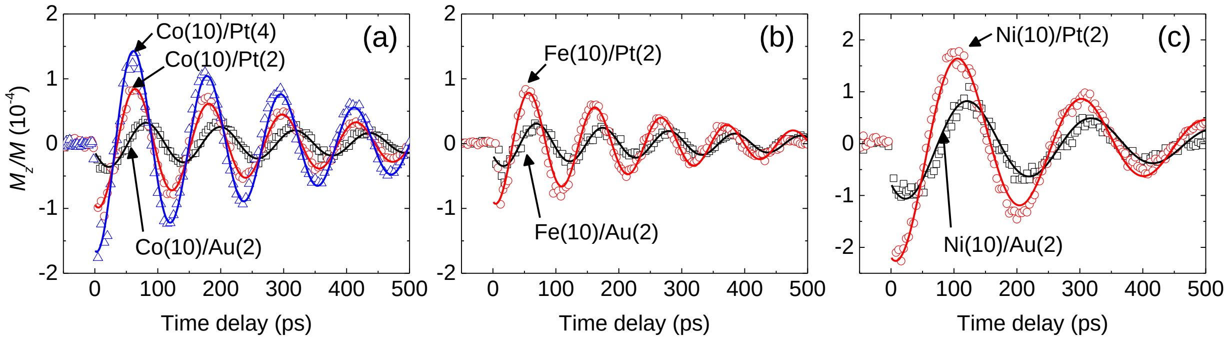

Supplementary Figure 2: The M_z dynamics of up to 500 ps (a) Data of Co(10)/Au(2) (black squares), Co(10)/Pt(2) (red circles), and Co(10)/Pt(4) (blue triangles). (b) Data of Fe(10)/Au(2) (black squares) and Fe(10)/Pt(2) (red circles). (c) Data of Ni(10)/Au(2) (black squares) and Ni(10)/Pt(2) (red circles). Solid lines are obtained by Landau-Lifshitz-Gilbert equation with initial magnetization tilting of Figure 4 of the main text, and precession frequency and damping constants of Supplementary Table 1.

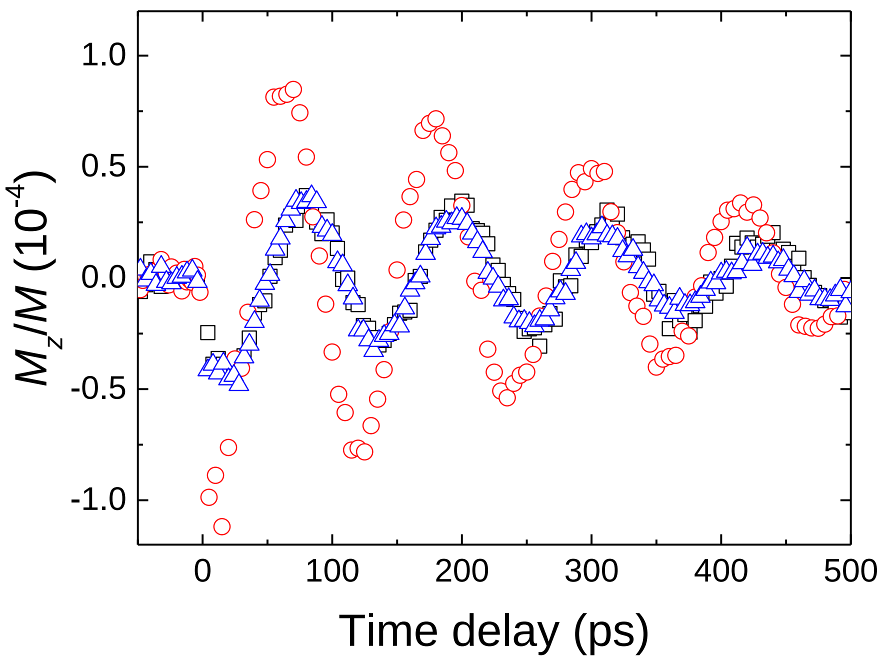

Supplementary Figure 3: The M_z dynamics of Co with MgO capping Data of the Co(10)/Au(2) (black squares), Co(10)/Pt(2) (red circles), and Co(10)/MgO(5) (blue triangles) samples.

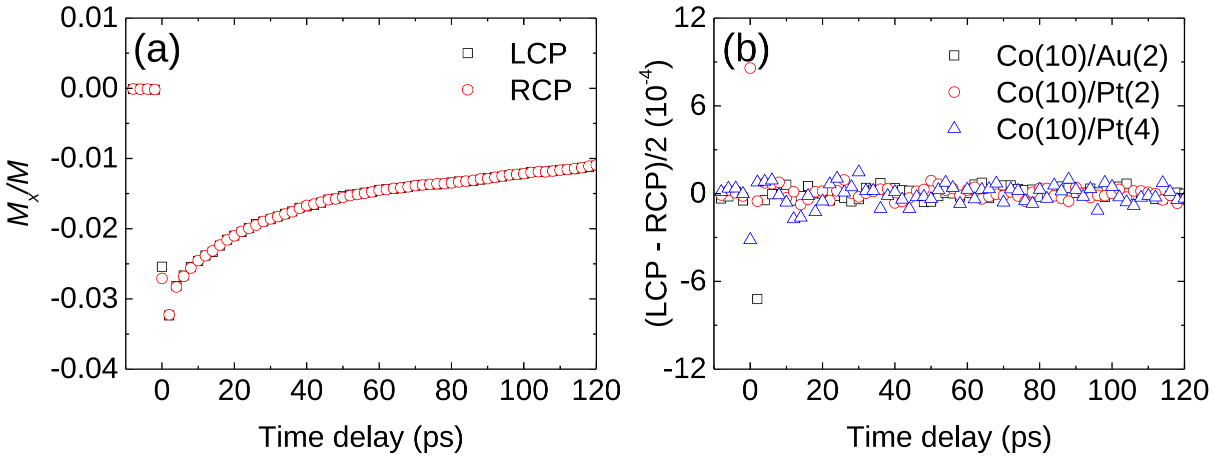

Supplementary Figure 4: The M_x dynamics Co with different capping layer (a) Data of the Co(10)/Pt(2) sample with LCP (black squares) and RCP (red circles). (b) Difference between LCP and RCP of the Co(10)/Au(2) (black squares), Co(10)/Pt(2) (red circles), and Co(10)/Pt(4) (blue triangles) samples.

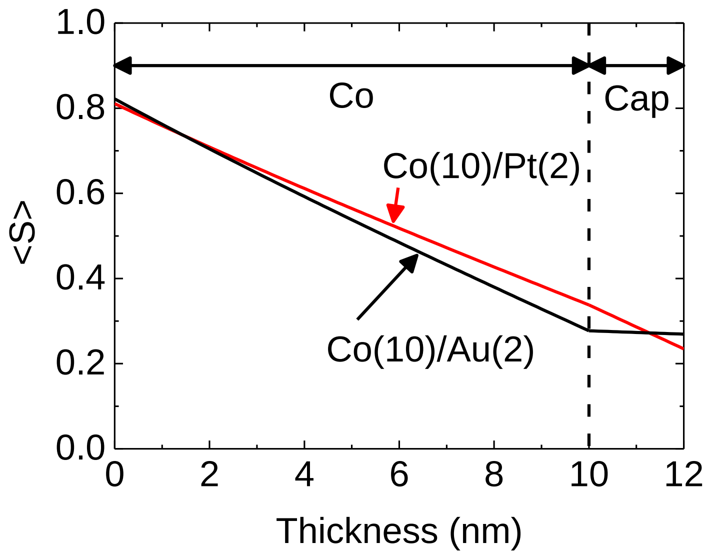

Supplementary Figure 5: The time-averaged Poynting vector through thickness Calculation for the sapphire substrate/Co(10)/Au(2) (black line) and sapphire substrate/Co(10)/Pt(2) structure with an incident Poynting vector of one. The Co layer lies from 0 to 10 nm, and the capping layer, Au or Pt, lies from 10 to 12 nm.

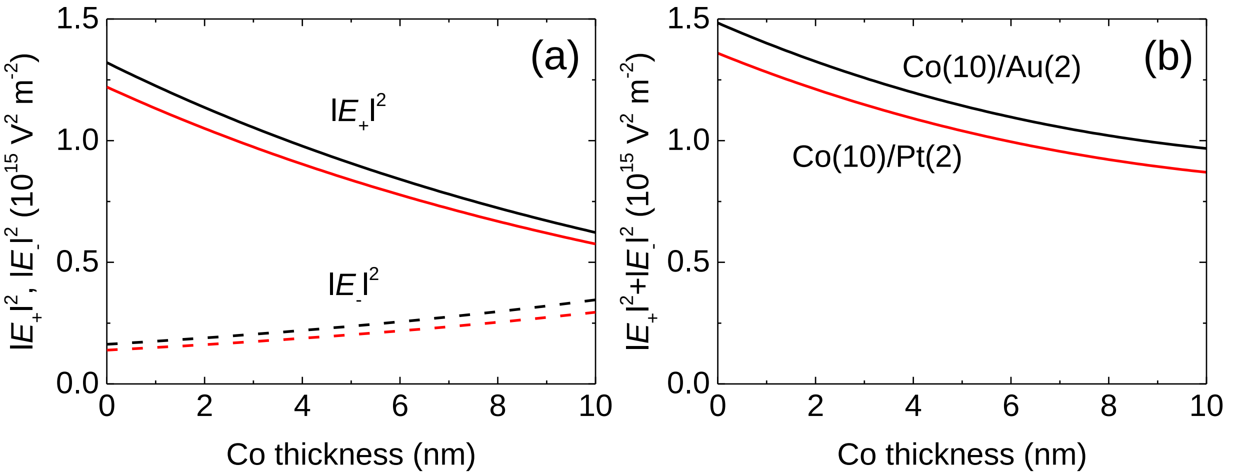

Supplementary Figure 6: The magnitude of electric field of light inside Co Calculation for the sapphire substrate/Co(10)/Au(2) (black lines) and sapphire substrate/Co(10)/Pt(2) (red lines) structure. (a) The $|E_+|^2$ is to the transmitted light (solid lines) and $|E_-|^2$ is due to the reflected light (dashed lines). (b) The sum of $|E_+|^2$ and $|E_-|^2$ inside Co of the sapphire substrate/Co(10)/Au(2) (black lines) and sapphire substrate/Co(10)/Pt(2) (red lines) structure.

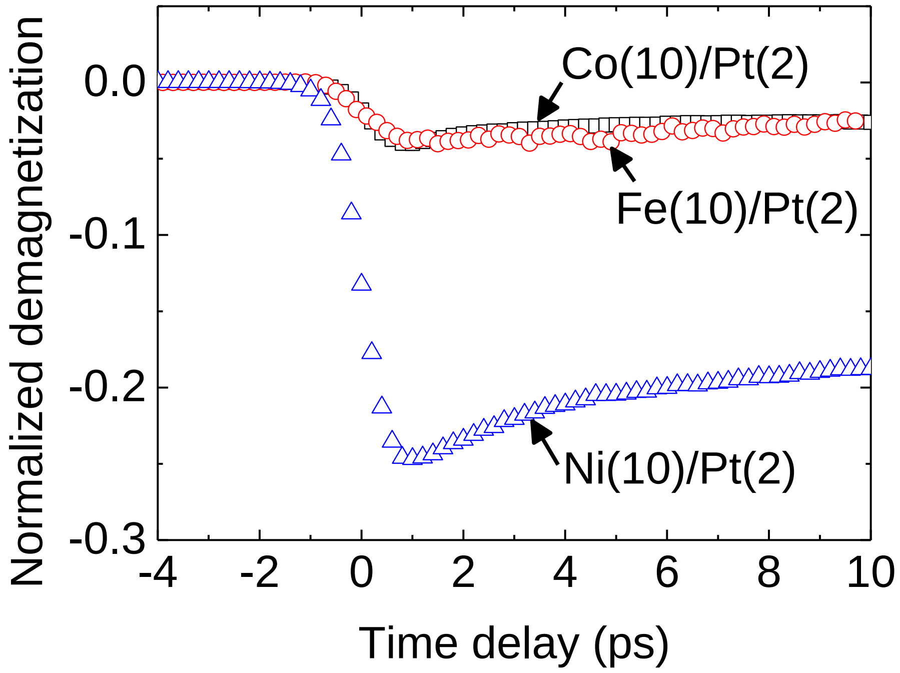

Supplementary Figure 7: The normalized demagnetization of FM Data with Co(10)/Pt(2) (black squares), Fe(10)/Pt(2) (red circles), and Ni(10)/Pt(2) (blue triangles) samples with incident fluence of 10 J m^{-2}.

Supplementary Table 1: The determination of the precession frequency (*f*) and damping constants (*α*) of all samples of Supplementary Figure 3.

	Co(10)/Au(2)	Co(10)/Pt(2)	Co(10)/Pt(4)	Fe(10)/Au(2)	Fe(10)/Pt(2)	Ni(10)/Au(2)	Ni(10)/Pt(2)
f (GHz)	8.5			9.6		5.0	
α	0.02	0.025	0.025	0.02	0.025	0.05	0.06

Supplementary References

1. M. van Kampen, C. Jozsa, J. T. Kohlhepp, P. LeClair, L. Lagae, W. J. M. de Jonge, and B. Koopmans, All-Optical Probe of Coherent Spin Waves, *Phys. Rev. Lett.* **88**, 227201 (2002).
2. Schellekens, A. J., Kuiper, K. C., de Wit, R. R. J. C., & Koopmans, B. Utrafast spin-transfer torque driven by femtosecond pulsed-laser excitation. *Nature Commun.* **5**, 4333 (2014).
3. Choi, G.-M., Min, B.-C., Lee, K.-J., & Cahill, D. G. Spin current generated by thermally driven ultrafast demagnetization. *Nature Commun*. **5**, 4334 (2014).
4. Johnson, P. B. & Christy, R. W., Optical constants of transition metals: Ti, V, Cr, Mn, Fe, Co, Ni, and Pd. *Phys. Rev. B* **9**, 5056-5070 (1974).
5. Rakić, A. D., Djurišić, A. B., Elazar, J. M., and Majewski, M. L., Optical properties of metallic films for vertical-cavity optoelectronic devices, *Appl. Opt.* **37**, 5271 (1998).
6. Hansteen, F., Kimel, A., Kirilyuk, A., & Rasing, Th., Femtosecond photomagnetic switching of spins in ferrimagnetic garnet films. *Phys. Rev. Lett.* **95**, 047402 (2005).
7. Choi, G.-M., Moon, C.-H., Min, B.-C., Lee, K.-J., & Cahill, D. G., Thermal spin-transfer torque driven by the spin-dependent Seebeck effect in metallic spin-valves. *Nature Phys.* **11**, 576-581 (2015).
